# MRI of Finger Pulleys at 7T—Direct Characterization of Pulley Ruptures in an Ex Vivo Model

**DOI:** 10.3390/diagnostics11071206

**Published:** 2021-07-03

**Authors:** Rafael Heiss, Alexander Librimir, Christoph Lutter, Rolf Janka, Stefanie Kuerten, Frank W. Roemer, Armin M. Nagel, Michael Uder, Thomas Bayer

**Affiliations:** 1 Institute of Radiology, University Hospital Erlangen, Friedrich-Alexander-Universität (FAU) Erlangen-Nürnberg, Maximiliansplatz 3, 91054 Erlangen, Germany; alex.librimir@gmx.de (A.L.); rolf.janka@uk-erlangen.de (R.J.); frank.roemer@uk-erlangen.de (F.W.R.); armin.nagel@uk-erlangen.de (A.M.N.); michael.uder@uk-erlangen.de (M.U.); thomas.bayer@klinikum-fuerth.de (T.B.); 2 Department of Orthopedics, University Medical Center, 18055 Rostock, Germany; christoph.lutter@googlemail.com; 3 Institute of Neuroanatomy, Medical Faculty, University of Bonn, 53115 Bonn, Germany; stefanie.kuerten@uni-bonn.de; 4 Institute of Anatomy and Cell Biology, FAU Erlangen-Nürnberg, Krankenhausstraße 9, 91054 Erlangen, Germany; 5 Department of Radiology, Boston University School of Medicine, Boston, MA 02118, USA; 6 Division of Medical Physics in Radiology, German Cancer Research Centre (DKFZ), 69120 Heidelberg, Germany; 7 Institute of Neuroradiology and Radiology, Klinikum Fürth, Jakob-Henle-Str. 1, 90766 Fürth, Germany

**Keywords:** finger flexor pulleys, ultra-high-field MRI, imaging, injury classification, climbing

## Abstract

The aim of this study was to evaluate 7 Tesla (7T) magnetic resonance imaging (MRI) for direct visualization and specific characterization of the finger flexor pulleys A2, A3, and A4 before and after ex vivo pulley rupture. Thirty fingers of human cadavers were examined before and after pulley disruption with a 26 min clinical 7T pulse sequence protocol. Images were assessed by two experienced radiologists for the presence of pulley rupture. Injury characterization included definition of rupture location, morphology, and complications. Image quality was evaluated according to a 4-point Likert-type scale from “not evaluable” to “excellent”. Macroscopic preparations were used as the reference standard. Direct characterization of intact A2, A3, and A4 pulleys and the corresponding pulley lesions was possible in all cases. The rupture location was distributed equally at the radial, ulnar, and central parts of the pulleys. A dislocation and intercalation of the pulley stump between the flexor tendon and finger phalanges was observed as a complication in 62.5% of cases. The average Likert score for direct visualization of pulleys was 2.67 before rupture and 2.79 after rupture creation, demonstrating adequate image quality for routine application. 7T MRI enables a direct characterization of A2, A3, and A4 pulleys before and after artificial disruption, including the definition of rupture morphology and location as well as the detection of rupture complications. This promises a precise presurgical evaluation of pulley injuries and complicated pulley stump dislocations.

## 1. Introduction

In recent years, magnetic resonance imaging (MRI) has gained importance as an imaging modality for specific diagnosis of soft-tissue trauma of the fingers. Improved dedicated protocols, especially those with ever-higher magnetic field strength, facilitate the correct diagnosis of ligamentous injuries, including the pulley system [1]. Diagnosis of pulley rupture has become particularly clinically relevant due to the increasing popularity of climbing in both recreational and professional athletes [2,3]. Finger pulley lesions are among the most common injuries in this new Olympic discipline, but they are also observed in non-climbers when high-impact forces act on an inflected finger [2,4,5]. Correct diagnosis of these injuries is necessary for therapy planning, as the pulley system is essential for unrestrained finger flexion. Untreated pulley lesions can cause chronic inflammation and a reduction in the range of motion, eventually leading to contractures [6,7]. Direct visualization of finger pulleys remains challenging in the radiological routine due to the small size of the anatomical structures involved [8]. Therefore, diagnostic methods applying ultrasound (US) or MRI are usually based on indirect evaluation of pulley ruptures via measurement of an increased, pathologic distance between finger flexor tendons and adjacent bone, referred to as bowstringing [8]. However, this indirect approach may be insufficient to differentiate between isolated and combined pulley ruptures [9]. In addition, direct visualization of the biomechanically relevant but small finger flexor pulley A3, which is typically involved in combined injuries, may not be possible with conventional MRI techniques [10]. As combined pulley injuries with A3 ligament involvement are commonly treated surgically, a correct diagnosis of such lesions is paramount [4,10]. Furthermore, improving imaging might also result in better detection of potential injury complications affecting prognosis, such as intercalation of a dislocated ruptured pulley stump between the flexor tendon and the bony cortex [11].

Recently, ultra-high-field MRI at 7T became clinically available for routine investigation, enabling imaging with high spatial resolution [12]. The purpose of this study was to investigate the feasibility of using 7T MRI with a clinical protocol for the direct depiction and characterization of the finger flexor pulleys A2, A3, and A4 before and after pulley injury in an ex vivo cadaveric model and to compare the results with those obtained from macroscopic and histopathological preparations.

## 2. Materials and Methods

### 2.1. Preparation of the Specimens

Thirty fresh frozen fingers (index finger, middle finger, and ring finger) from ten paired hands were obtained from three female and two male cadavers donated to the Institute of Anatomy, Friedrich-Alexander-University Erlangen-Nuremberg, Germany. Institutional Review Board (IRB) approval was obtained from Friedrich-Alexander-University Erlangen-Nuremberg, Germany (260_15 Bc). All participants had signed an institutionally approved informed consent document for being a body donor. Donors had a mean age of 77.4 years (range 55–94 years) at time of death. After an intermediate cooling to 5 °C for a maximum of two days, the forearms were harvested and stored at −15 °C. Each specimen was prepared after de-freezing according to the protocol described by Schöffl et al. [13]. The tendons of the flexor digitorum superficialis (FDS) muscle and the flexor digitorum profundus (FDP) muscle were prepared proximal to the fingers and were intersected at the myotendinous junction at the forearm. The index finger (Dig. 2), middle finger (Dig. 3), and ring finger (Dig. 4) were extra-articulated at the carpometacarpal level, and two Schanz screws were placed within the metacarpal bone for later fixation in a loading apparatus. Fingers were placed in crimp grip position (a typical climbing position associated with pulley rupture) in a custom-made loading apparatus [14,15,16]. The finger flexor pulley ruptures were induced by loading the FDS and FDP tendons of the fingers using an isokinetic loading device [9,17]. The flexor tendons were connected in series with the force transducers and the electric cage motor as illustrated in a previous study [13]. By doing so, we were able to increase the forces in the flexor tendons while the finger remained stationary, performing a concentric movement. Loading of the tendons continued until failure. Failure reasons observed were pulley rupture (noticeable typical acoustic bang), fracture of bone, tendon rupture, or no inducible anatomical injury [13].

### 2.2. Magnetic Resonance Imaging

**Figure 1:**
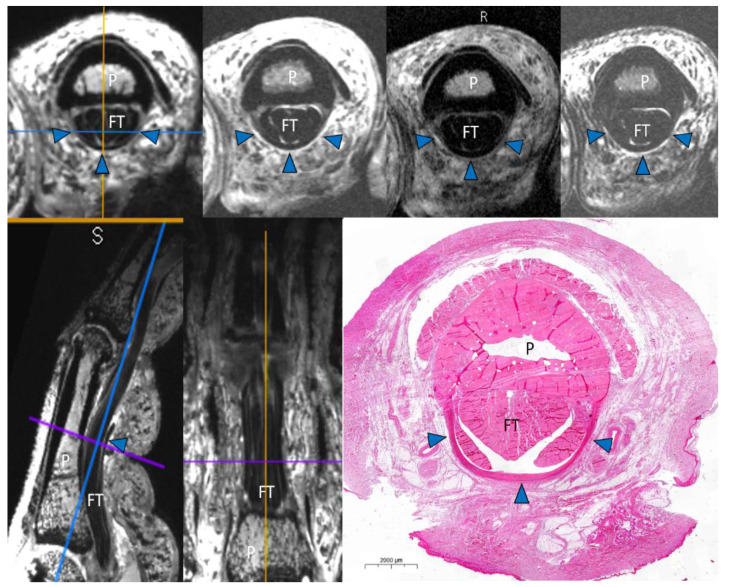
Example of the study protocol with an intact A2 pulley. Three-dimensional double echo steady-state (3D DESS) axial multiplanar reconstruction (MPR), axial PD fs, T1 turbo spin echo (TSE), and T2 TSE scans (**above**). 3D DESS sagittal and coronal MPR. Histological correlation (**below**). P = phalanx. FT = flexor tendon. Arrowheads = intact A2 pulley.

MRI examinations were performed on a 7T scanner (MAGNETOM Terra, Siemens Healthcare, Erlangen, Germany) using a prototype 1-channel transmit/16-channel receive wrist radiofrequency coil (Rapid Biomedical GmbH, Rimpar, Germany) before and after the induction of artificial pulley injuries. The 16-channel receive array had an elliptical cross-section (78 mm × 98 mm) and a length of 70 mm. Fingers were placed in a cast with 30-degree flexion within the proximal interphalangeal (PIP) joint. A force of 10 N was applied by connecting a weight of 1.0 kg through a pulley rope system to the FDS and FDP tendons, with equal distribution to both tendons [9]. A transverse T1-weighted turbo spin echo (TSE) sequence (total acquisition time, 4 min 46 s; echo time, 17 ms; repetition time, 700 ms; resolution, 0.2 × 0.2 × 1.5 mm), a transverse T2-weighted TSE sequence (total acquisition time, 6 min 32 s; echo time, 68 ms; repetition time, 5000 ms; resolution, 0.2 × 0.2 × 1.5 mm), a transverse PD-weighted TSE sequence (total acquisition time, 6 min 32 s; echo time, 14 ms; repetition time, 5640 ms; resolution, 0.2 × 0.2 × 1.5 mm), and a three-dimensional double echo steady-state (3D DESS; total acquisition time, 8 min 55 s; echo time, 6.38 ms; repetition time, 17.97 ms; flip angle, 21°; resolution, 0.3 × 0.3 × 0.3 mm) were acquired (Figure 1).

### 2.3. Image Analysis

All MRIs were reviewed to assess the overall image quality and the type of finger flexor pulley injury by two musculoskeletal radiologists with 12 and 15 years of experience, respectively (T.B. and F.R.). Those scoring the MRI images were blinded to the reports of the artificial injury induction and to the macroscopic analysis. The image quality of each finger flexor pulley (A2, A3, and A4) was evaluated on a 4-point Likert-type scale from insufficient to excellent: 0—insufficient (visibility of pulley heavily degraded due to insufficient spatial resolution, signal intensity, or artifacts, with no information about the pulley); 1—poor (visibility of pulley degraded due to limitation of spatial resolution, signal intensity, or disturbance by artifacts, with poor information about the pulley visibility); 2—good (sufficient spatial resolution and signal intensity with only slight artifacts, resulting in a visualization of normal pulleys > 90% of their circumference); and 3—excellent (sufficient spatial resolution and signal intensity without disturbance by artifacts, resulting in clear and sharp depiction of entire normal pulley). A consensus reading to select a matching rating for image quality and rupture characterization was performed in cases of discrepancy between the radiologists. Rupture patterns of each A2, A3, and A4 finger flexor pulley were graded individually by each radiologist as follows: grade 0 (NO.RUPT)—intact, grade 1 (SMOOTH.RUPT)—rupture with stump visible adjacent to the flexor tendon, grade 2 (DISLOC.RUPT)—rupture with intercalation (dislocation of the pulley stump between the flexor tendon and finger phalanx), grade 3 (PULLEY.LOST)—rupture without visible stump, and grade 4 (N.A.)—not assessable. In the presence of a finger flexor pulley injury, the location of the rupture was graded according to the anatomical position: r—rupture on the radial side of the finger flexor pulley insertion with the stump of the finger flexor pulley visible on the ulnar side; u—rupture on the ulnar side of the finger flexor pulley insertion with the stump of the finger flexor pulley visible on the radial side; and m—rupture in the middle of the finger flexor pulley with the stump of the finger flexor pulley visible on both sides.

### 2.4. Macroscopic and Histopathologic Inspection

After MRI scans, all finger flexor pulleys were anatomically prepared by a specialized orthopedic surgeon (C.L.) with five years of experience in finger and wrist surgery and inspected for integrity or injury using magnifying glasses. Rupture patterns were determined to be analogous to radiological assessment by an orthopedic surgeon and recorded by photo documentation. For additional comparison of microstructural anatomic findings, histological slices were cut frozen in an axial plane. Hematoxylin and eosin staining was performed using standard techniques. Scanning and digitalization of the slides were performed using dedicated software (Case Viewer, 3DHISTECH, Budapest, Hungary).

## 3. Results

MRI scans before and after trauma were acquired for all 30 cadaver fingers. The consensus reading with direct pulley characterization revealed a total of 22 flexor pulley ruptures (out of a possible 90) in 11 of the 30 fingers. These ruptures were equally observed in left (11/22) and right (11/22) hands. The rupture occurred at an index finger in 59% of the cases (13/22), at a middle finger in 23% of cases (5/22), and at a ring finger in 18% of cases (4/22). The existing rupture patterns included five triple ruptures (A2/A3/A4), one combined rupture (A2/A3), and five single ruptures (3 A2, 2 A4). Finger flexor A2, A3, and A4 pulley lesions were detected at the radial, ulnar, and central parts of the finger pulley, with 33.3% of the lesions occurring in each of these locations. A dislocation and intercalation of the pulley stump grade 2 (DISLOC.RUPT) between the flexor tendon and finger phalanx was observed in 62.5% of the pulley lesions. MRI findings were confirmed by anatomical inspections in all cases. An overview of the distribution of finger flexor pulley ruptures is given in Table 1.

### Image Analysis

**Figure 2:**
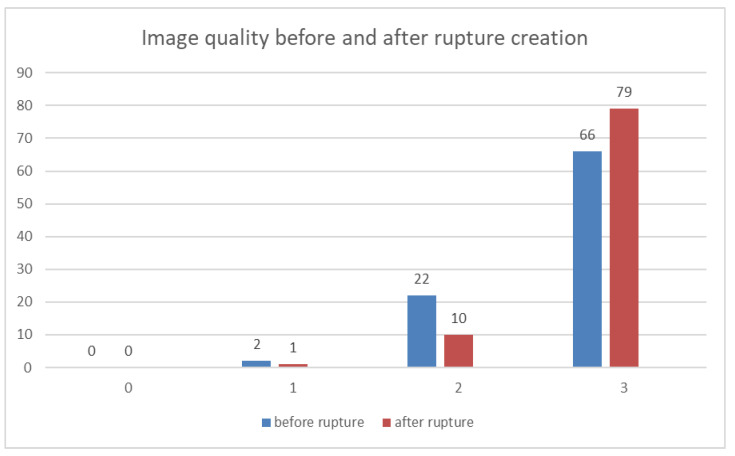
Image quality for the visualization of finger flexor pulleys before and after rupture creation evaluated according to a 4-point Likert-type scale from not evaluable (0) to excellent (3), separated by the two reviewers in consensus. No specimen was rated as having a not-evaluable image quality (0).

The average Likert score for direct visualization of finger flexor pulleys was 2.67 before rupture and 2.79 after rupture, reflecting adequate image quality on average. The visualization of each pulley separately revealed a Likert score of 2.74 for A2 and 2.64 for both A3 and A4 finger flexor pulleys (Figure 2).

**Figure 3:**
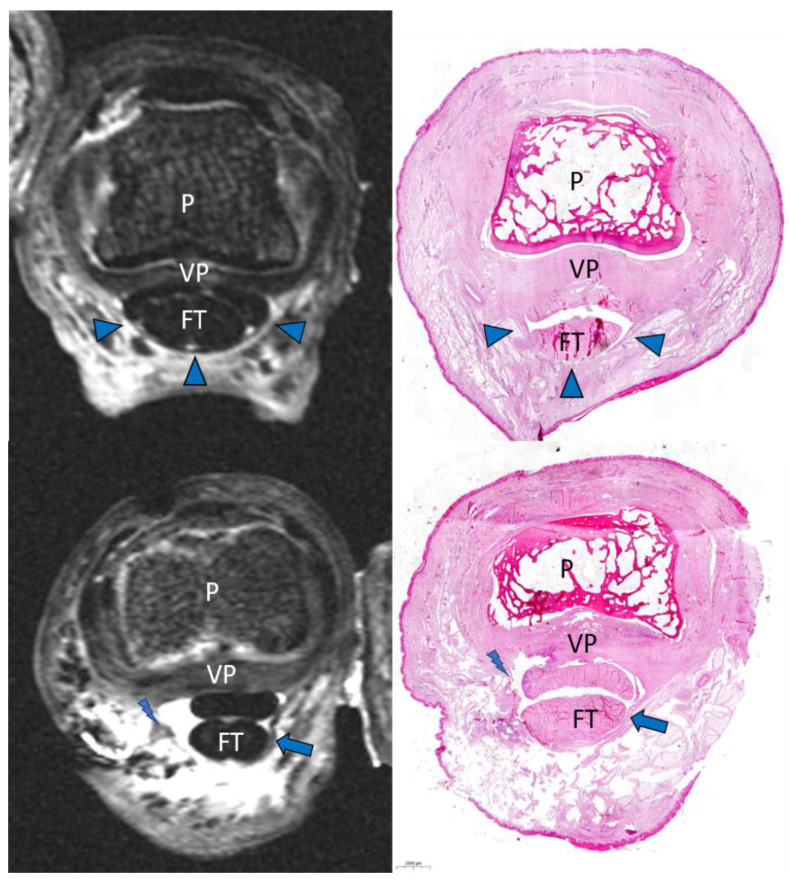
Comparison of an A3 pulley before and after rupture. Axial PD fs and histologic correlation before rupture creation (**above**) and after rupture creation (**below**). P = phalanx. This A3 rupture was characterized and confirmed as grade 1 (SMOOTH.RUPT). FT = flexor tendon. Arrowhead = intact A3 pulley. Arrow = disrupted pulley stump. Lightning bolt = exact spot of rupture. VP = volar plate.

**Figure 4:**
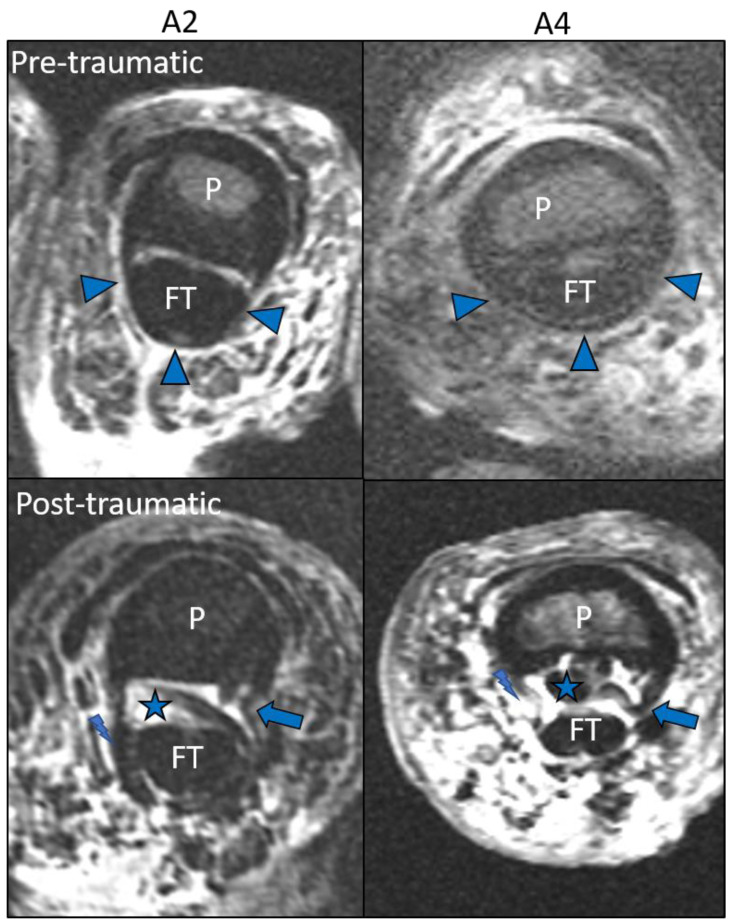
Characterization of pulley rupture with definition of intercalation as an injury complication. PD fs axial scans before (**above**) and after (**below**) iatrogenic pulley rupture creation. These A2 and A4 ruptures were characterized and confirmed as grade 2 (DISLOC.RUPT). Left A2, right A4 pulley. P = phalanx. FT = flexor tendon. Arrowhead = intact A2 and A4 pulley. Arrow = disrupted pulley stump. Asterisk = intercalation of disrupted pulley stump between FT and P. Lightning bolt = exact spot of rupture.

Overall agreement in identifying finger flexor pulleys and ruptures was 99%. One discrepant rupture characterization occurred in an A3 pulley of a specimen that was rated with an image quality of grade 1 (insufficient) by both observers. This pulley was characterized as grade 3 (PULLEY.LOST) in the subsequent consensus reading with confirmation in the anatomical inspection. All intercalations of the pulley stumps between the flexor tendon and finger phalanx were detected in MRI by both observers. Example images of the injuries observed are presented in Figure 3 and Figure 4.

## 4. Discussion

We evaluated ultra-high-field MRI at 7T for direct visualization of intact and disrupted finger flexor pulleys using a clinical scan protocol of less than 30 min duration. The resulting image quality enabled direct characterization of all traumatic pulley lesions, including rupture morphology, rupture pattern, and rupture location, even for the smaller pulleys A3 and A4. It allowed definition of potentially relevant trauma complications, such as intercalation of ruptured pulley stumps with dislocation between the flexor tendons and the phalanx.

It is expected that direct characterization of pulley ruptures will become more common in the clinic in the future due to the increasing popularity of climbing as a new Olympic discipline [2,5]. Clinical examination has proven to be inaccurate in the acute phase when edema is still present [1,3]. Radiographs and CT can only demonstrate nonspecific soft tissue swelling or the “bowstring sign”; due to their low contrast resolution, they cannot directly show the pulleys [6]. Ultrasound (US) and conventional MRI at 1.5T or 3T are currently the reference standard imaging modalities using typically indirect approaches, but both techniques have shortcomings. The commonly used “bowstring sign” is based on measurements of an augmented distance between the flexor tendons and the adjacent phalanx (TB), but cut-off points for distinguishing single A2 pulley ruptures from combined A2/A3 pulley ruptures via TB measurements are inconsistent [8,18,19]. A possible explanation for ambiguous results is that the exact anatomic definition of TB measurement, the angles over the PIP joints, and the forces on the flexor tendons are not standardized [9,10]. Direct finger flexor pulley MRI is not susceptible to this drawback, but from a technical point of view, it is more challenging than classical indirect stress imaging due to the extremely small size of the delicate pulley structures, especially for the smallest pulley (A3) [8]. Therefore, imaging at lower magnetic field strength may frequently yield an unsatisfactory direct depiction of disrupted pulleys, resulting in unclear definition of the injury pattern. Few studies have investigated direct pulley imaging, with two studies exclusively addressing the A2 pulley at 3T MRI [6]. A further increase in magnetic field strength, as in our study, may be used to reduce the scan time and/or to improve spatial resolution compared to lower magnetic field strength. In addition, a dedicated finger radiofrequency coil could be used to further improve spatial resolution [20], as this enables a superior visualization of smaller anatomical structures and may enable diagnosis of pathologies previously not assessable [21]. This may improve the consistency in differentiating between isolated and combined pulley ruptures [9,22] when imaging is performed in a neutral prone position such as in our ultra-high-field protocol. Hauger et al. and Bencardino emphasized that the transverse plane proved to be more reliable than the sagittal plane for pulley depiction and offered optimal visualization of the bony insertions of the pulleys [8,23]. Therefore, our study protocol included three sequences in transversal planes with all fingers scanned in a neutral position. In addition, a high-resolution 3D DESS sequence was acquired in all specimens for further analysis of microstructural pulley details using multiplanar reconstructions. To our knowledge, this is the first study describing the normal and posttraumatic appearance of the functionally important pulley A3 in MRI [24,25].

Furthermore, an important finding of our study was the proof of dislocated ruptured pulley stumps between the flexor tendon and finger phalanges. The resulting mechanical complication might correlate with underdiagnosed clinical cases with prolonged recovery and chronic tenosynovitis, such as those described as a flap irritation phenomenon (FL.I.P) after minor pulley rupture by Schöffl et al. in 2011 [11]. A reliable direct visualization of these dislocated pulley stumps may influence therapeutic decision making and contribute to surgery indication. Correct diagnosis of this complication could improve prognosis for individuals who would suffer from a prolonged recovery with inflammatory pain and likely not fully regain finger function due to delayed surgery [11].

Moreover, ultra-high-field MRI enabled definition of the exact anatomic morphology and location of all pulley ruptures. In our study, this allowed analysis of the rupture morphology and evaluation of the rupture side distribution, which was homogeneously divided into the radial, ulnar, and middle parts of the finger pulleys in our sample. In a clinical context, a reliable and ideally direct visualization of the disrupted finger flexor pulley ligaments might improve therapy planning [4,10]. Differentiating partial from complete single ruptures would allow a more precise planning of conservative treatment regimens adopting immobilization and better estimation of recovery times. In cases with complete multiple pulley rupture, which are usually treated surgically, numerous surgical pulley reconstruction techniques have been described in the literature [23,26,27,28,29,30]. For these cases, direct imaging could contribute to a better presurgical determination of the appropriate reconstruction and planning of the suture and/or graft technique.

Our study has several limitations. Implications from a cadaver study must be translated cautiously to clinical settings due to potential differences in the biomechanical characteristics of post-mortal tissue and due to the artificial lesion induction in our study. Due to the rapid freeze–thaw cycle, changings of tissue properties seem of lesser importance for our study. We tried to mimic realistic ruptures by tendon overloading without influencing the pulleys by incision. Nevertheless, some of our specimens were impaired by artifacts, which primarily resulted from air entrapment between tendon and bone. It must also be stressed that all fingers included were from an elderly population, which is in contrast with the younger mean age of active athletes. We designed this pilot study to demonstrate the feasibility of a single 7T system sufficient to characterize pulley ruptures using direct images without comparing the results to other image modalities. Therefore, a calculation of sensitivity and specificity values compared with histopathological and anatomical analysis (the gold standard) were not the aim of this study. We expect that further coil optimization will lead to better image quality, even at field strengths other than 7T. Thus, analysis of sensitivity and specificity compared to other imaging modalities and MRI field strengths is planned in future studies.

## 5. Conclusions

Ultra-high-field MRI at 7T is a promising method for direct injury depiction of the finger flexor tendon pulleys and may improve diagnostic confidence. This may contribute to improved clinical decision making and patient outcomes by guiding appropriate treatment strategies, especially in cases with combined pulley ruptures or rupture complications such as intercalation of pulley stumps. Our study suggests that 7T MRI may improve diagnosis of finger injuries, as it is a high-end musculoskeletal application with the ability to define delicate lesions, which is not achievable at lower field strengths.

## Figures and Tables

**Table 1:** Macroscopic distribution of finger flexor pulley A2, A3, and A4 ruptures at the index finger (2), middle finger (3), and ring finger (4) separated by side (L—left; R—right) and characterized by rupture morphology (0 (NO.RUPT)—intact finger flexor pulley, 1 (SMOOTH.RUPT)—rupture with stump visible adjacent to the flexor tendon, 2 (DISLOC.RUPT)—rupture with intercalation (dislocation of the pulley stump between the flexor tendon and finger phalanx), and 3 (PULLEY.LOST)—rupture without visible stump; side of finger flexor pulley rupture, r—radial, u—ulnar, m—middle).

		Characterization of Pulley Rupture		
Specimen	Finger	A2	A3	A4
1	L2	1(SMOOTH.RUPT)r	0(NO.RUPT)	0(NO.RUPT)
1	L3	2(DISLOC.RUPT)m	3(PULLEY.LOST)	2(DISLOC.RUPT)u
1	L4	2(DISLOC.RUPT)u	2(DISLOC.RUPT)u	0(NO.RUPT)
2	L2	2(DISLOC.RUPT)m	2(DISLOC.RUPT)m	2(DISLOC.RUPT)u
2	L3	0(NO.RUPT)	0(NO.RUPT)	2(DISLOC.RUPT)u
2	L4	0(NO.RUPT)	0(NO.RUPT)	2(DISLOC.RUPT)u
2	R2	2(DISLOC.RUPT)r	2(DISLOC.RUPT)m	2(DISLOC.RUPT)u
3	R2	2(DISLOC.RUPT)u	1(SMOOTH.RUPT)m	2(DISLOC.RUPT)r
3	R3	1(SMOOTH.RUPT)r	0(NO.RUPT)	0(NO.RUPT)
4	R2	2(DISLOC.RUPT)r	2(DISLOC.RUPT)r	2(DISLOC.RUPT)r
5	R4	2(DISLOC.RUPT)r	0(NO.RUPT)	0(NO.RUPT)
Total		9	6	7

## Data Availability

Not applicable.
